# Construction and validation of nomogram prediction model for anxiety and depression in chemotherapy patients with multiple myeloma

**DOI:** 10.3389/fpsyt.2025.1578132

**Published:** 2026-01-12

**Authors:** Liping Wu, Yizhen Li, Hui Li

**Affiliations:** 1 Department of pharmacy, Ganzhou People’s Hospital, Ganzhou, Jiangxi, China; 2 Department of Oncology & Hematology, Guangzhou Hospital of Integrated Traditional and Western Medicine, Guangzhou, Guangdong, China; 3 Department of Hematology, The Second Affiliated Hospital of Guangzhou University of Chinese Medicine, Guangzhou, Guangdong, China

**Keywords:** multiple myeloma, chemotherapy, anxiety and depression, nomogram, influence factor

## Abstract

**Objective:**

To construct and validate a nomogram prediction model for anxiety and depression in chemotherapy patients with multiple myeloma (MM).

**Methods:**

From May 2021 to May 2023, 333 MM chemotherapy patients treated at our hospital were selected. The patients were randomly divided into a modeling group and a validation group in a 7:3 ratio (using a random number table method). According to whether the modeling group patients had anxiety and depression, they were grouped into a non-anxiety and depression group and an anxiety and depression group; a nomogram model constructed using R software was built to evaluate the predictive performance of the model. Clinical decision curves (DCA) were used to assess the value of the clinical application of the model.

**Results:**

Out of the 233 patients in this study, 66 experienced anxiety and depression, with an incidence rate of 28.33%. There were great differences in gender, economic income, education level, tumor staging, complications, and treatment plans between the anxiety and depression group and the non-anxiety and depression group (P<0.05). The results of multivariate Logistic regression analysis showed that gender, education level, tumor stage, complications, and treatment plan were risk factors for anxiety and depression in MM chemotherapy patients (P<0.05), while economic income served as a protective factor (P<0.05). The AUC of the modeling group was 0.945, and the H-L test was χ2 = 8.579, P = 0.674, with good agreement. The AUC of the validation group was 0.967, and the H-L test was χ2 = 7.315, P = 0.698, with good consistency. The DCA curve shows that the probability of assessing anxiety and depression in patients undergoing MM chemotherapy is higher for clinical use when the probability is between 0.05 and 0.95.

**Conclusion:**

Gender, education, tumor stage, comorbidities and treatment regimen were risk factors for anxiety and depression in MM chemotherapy patients, while financial income was a protective factor for anxiety and depression in MM chemotherapy patients, and the constructed nomogram visually predicted the risk of anxiety and depression in MM chemotherapy patients.

## 1 Introduction

Multiple Myeloma (MM) is a common clinical malignancy, most frequently seen in middle-aged and elderly people. MM can suppress the body’s immune function, increasing the risk of bacterial infections, and the body is also prone to kidney damage, hypercalcemia, all of which have a serious impact on the patient’s health (1, 2). Clinical chemotherapy is an important method for treating MM, which can effectively improve the prognosis of patients and extend their survival time. However, during the chemotherapy process, while the drugs kill tumor cells, they may also damage normal cells, causing patients to experience various side effects such as nausea, vomiting, hair loss, which adversely affect physical and mental health, and are prone to anxiety and depression (3, 4). There are many factors affecting anxiety and depression in patients with malignant tumors. Promptly identifying and intervening in the relevant factors is particularly important for improving patient prognosis. A nomogram is a risk prediction model that converts influencing factors into numerical probabilities of clinical events, predicting the risk of occurrence and assisting clinicians in formulating preventive measures (5). A visual obesity risk prediction system based on machine learning has been developed, demonstrating good predictive performance and interpretability. The system directly provides users with their obesity risk level and identifies corresponding intervention priorities, helping physicians formulate personalized health management plans and achieving comprehensive and accurate obesity management (6). Based on the CHARLS cohort study, a visual risk prediction system for sarcopenia in the elderly was constructed using machine learning. This system supports early identification and scientific intervention of sarcopenia in older adults, with significant clinical value and application potential (7). A risk prediction system for depression in middle-aged and elderly individuals was also established using machine learning and visualization techniques. By providing early detection and evidence-based interventions for depression in this population, it establishes a new health management paradigm and has the potential to improve quality of life (8). Currently, there are few reports on the risk prediction research of anxiety and depression in MM chemotherapy patients, therefore, this study mainly aims to construct and verify a nomogram prediction model for anxiety and depression in MM chemotherapy patients.

## 2 Subjects and methods

### 2.1 Study subjects

**Figure 1:**
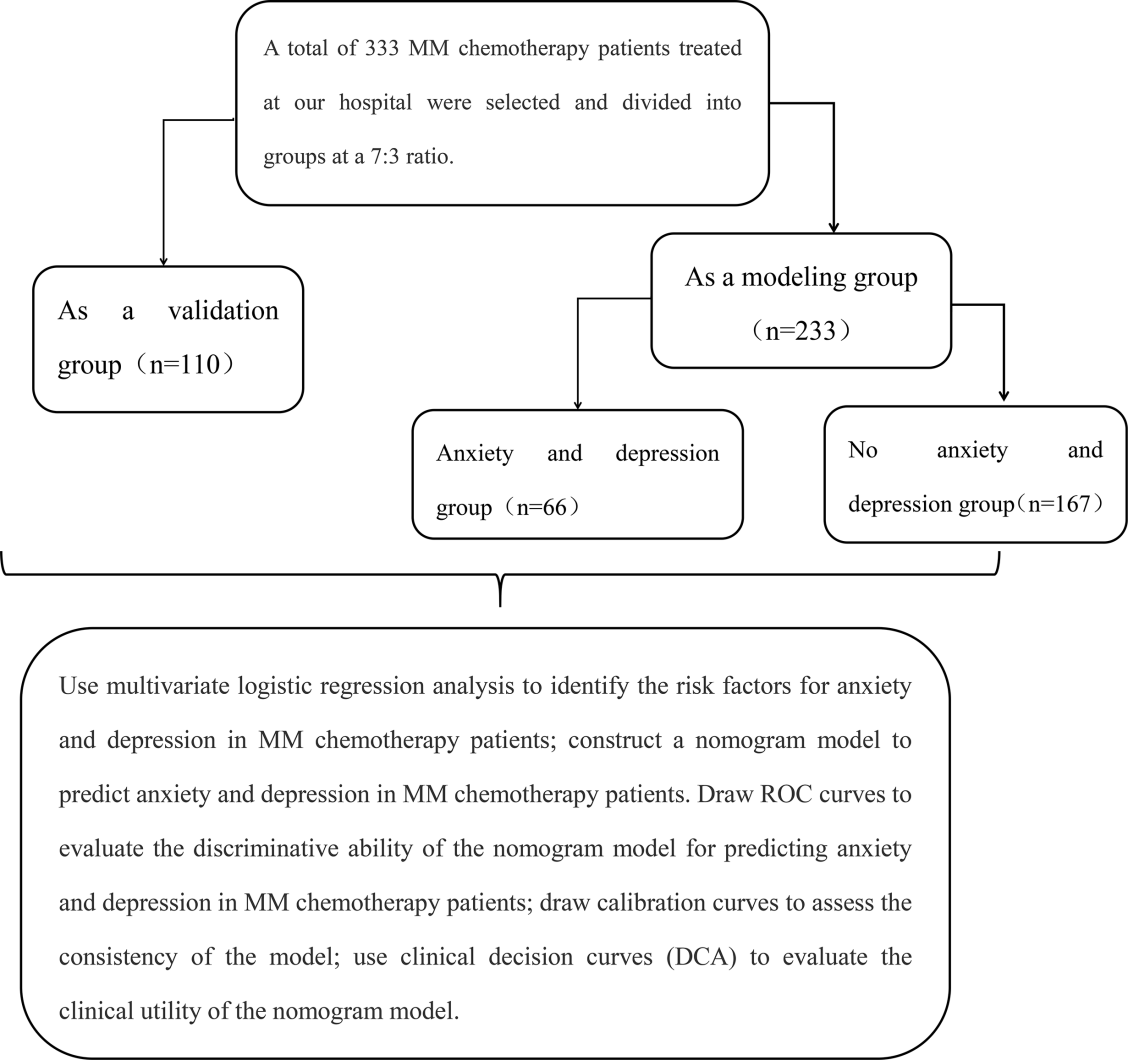
Flow chart of case collection.

333 MM chemotherapy patients treated in our hospital from May 2021 to May 2023 were selected, aged 54-86 years. Sample size calculation was performed using PASS 15 software (two-sided test, α = 0.05, power = 90%, d = 0.50), the total required sample size was calculated to be 301. Considering a 10% dropout rate, at least 333 participants need to be enrolled. The enrollees were randomly divided into a modeling group (233 cases) and a validation group (100 cases) at a 7:3 ratio (using a random number table method), and according to whether patients in the modeling group experienced anxiety and depression, they were divided into an anxiety-depression group and a non-anxiety-depression group. The case-selection flowchart is shown in **Figure 1**. Inclusion criteria: ① Conforming to the relevant diagnostic criteria for MM (9), confirmed by imaging; ② All receiving chemotherapy; ③ Stable condition, with no abnormal organ function (except for the kidneys); ④ Complete data. Exclusion criteria: ① Severe condition; ② Communication barriers; ③ History of alcohol or drug dependence; ④ Severe physical illness; ⑤ Stressful events occurring before admission. Patients signed a consent form, and this study was approved by the ethics committee of our hospital.

### 2.2 Criteria for assessing anxiety and depression in MM chemotherapy patients

Anxiety and depression are assessed using the Hospital Anxiety and Depression Scale (HADS) (10) (two subscales for anxiety and depression), each consisting of 7 items scored from 0 to 3. A total score of ≤7 points (no anxiety or depression) is classified as the non-anxiety and depression group, while scores of 8-10 points (possible anxiety or depression) and >10 points (definite anxiety or depression) are classified as the anxiety and depression group.

### 2.3 Collection of clinical data

Data collected includes patient’s age, Body Mass Index (BMI), gender, place of residence, economic income (grouped by the median value for statistical analysis), family support, marital status, educational level, method of medical expense payment, employment status, tumor staging, complications(anemia, bone pain, hyperviscosity syndrome, and renal impairment), treatment regimen (MPT regimen: Melphalan + Prednisone + Thalidomide; VDT regimen: Bortezomib + Dexamethasone + Thalidomide), coping strategies (assessed using a simple coping style questionnaire with a four-level scoring system, including positive and negative coping), and the Social Support Rating Scale (SSRS) to assess social support (with a maximum score of 62, higher scores indicating greater social support).

### 2.4 Method of data collection

All personnel in this study were uniformly trained to ensure consistent understanding of the questionnaire items. Data collection was mainly done through the hospital’s electronic medical record system on computer terminals, using specially designed pathology data collection forms. The data collectors, proficient in using the electronic medical record system and with over five years of experience and research capability, compiled the survey contents item by item to ensure the validity and authenticity of the data.

### 2.5 Statistical processing

Data were analyzed using SPSS 25.0. Count data were tested with the *χ*^2^ chi-square test and expressed as cases (%). Quantitative data (conforming to normal distribution) were tested with the t-test and expressed as $\bar{x}\pm s$ (mean ± standard deviation). Multifactorial logistic regression analysis (enter method) was used to analyze the risk factors for anxiety and depression in MM chemotherapy patients; The identified risk factors were then introduced into R3.6.3 software using the rms package to construct a nomogram model for predicting anxiety and depression in MM chemotherapy patients. The nomogram model was generated to visually represent the contribution of each independent variable to the predicted probability of anxiety and depression, allowing for an intuitive assessment of risk for each patient based on their clinical characteristics. ROC curves were drawn to evaluate the discriminative ability of the nomogram model for predicting anxiety and depression in MM chemotherapy patients; calibration curves were drawn to assess the consistency of the model. The clinical decision curve analysis (DCA) was used to evaluate the clinical application value of the nomogram model. P<0.05 was considered statistically significant.

## 3 Results

### 3.1 Anxiety status in MM chemotherapy patients

The results of this study show that 167 patients had no anxiety or depression, 41 possibly affected, and 25 had definite anxiety or depression. The depression score for the anxiety and depression group is (9.40 ± 1.30) points, and the anxiety score is (10.60 ± 1.40) points.

### 3.2 Comparison of clinical data between modeling and validation groups

There were no significant differences (P>0.05) in clinical data such as age, BMI, gender, place of residence, economic income, family support, marital status, educational level, method of medical expense payment, employment status, tumor staging, complications, treatment regimen, coping methods, and SSRS scores between the modeling and validation groups. Details are shown in **Table 1**.

**Table 1:** Comparison of clinical data between the modeling group and the validation group.

Factor	Modeling group (n=233)	Validation groups(n=100)	*t*/*χ*^2^	*P*
Age(year)			0.455	0.500
≥60	167(71.67)	68(68.00)		
<60	66(28.33)	32(32.00)		
BMI(kg/m^2^)	23.61 ± 4.28	23.59 ± 4.29	0.039	0.969
Gender			0.231	0.631
man	144(61.80)	59(59.00)		
woman	89(38.20)	41(41.00)		
Place of residence			0.284	0.594
town	147(63.09)	60(60.00)		
countryside	86(36.91)	40(40.00)		
Economic income (10,000 yuan/year)			1.003	0.317
≥5	135(57.94)	52(52.00)		
<5	98(42.06)	48(48.00)		
Family support situation			0.053	0.819
support	129(55.36)	54(54.00)		
Not supported	104(44.64)	46(46.00)		
Marital status			0.510	0.475
married	163(69.96)	66(66.00)		
Unmarried/divorced/widowed	70(30.04)	34(34.00)		
Educational level			0.176	0.675
Junior high school and below	106(45.49)	43(43.00)		
High school and above	127(54.51)	57(57.00)		
Payment methods for medical expenses			0.160	0.689
No health insurance	49(21.03)	23(23.00)		
Medical Health Insurance	184(78.97)	77(77.00)		
Working conditions			0.124	0.725
incumbency	137(58.80)	56(56.00)		
retire	96(41.20)	44(44.00)		
Tumor staging			0.177	0.674
I~II	127(54.51)	52(52.00)		
III	106(45.49)	48(48.00)		
Complication			0.139	0.710
Yes	103(44.21)	42(42.00)		
No	130(55.79)	58(58.00)		
Treatment regimen			0.018	0.892
MPT scheme	96(41.20)	42(42.00)		
VDT scheme	137(58.80)	58(58.00)		
Coping			0.104	0.747
Be proactive	121(51.93)	50(50.00)		
Negative coping	112(48.07)	50(50.00)		
SSRS Score (points)			0.089	0.765
<40	109(46.78)	45(45.00)		
≥40	124(53.22)	55(55.00)		

### 3.3 Comparison of clinical data between anxiety and depression group and non-anxiety and depression group

Statistically, among the 233 patients in this study, 66 had anxiety or depression, with an incidence rate of 28.33%. There were differences (P<0.05) in gender, economic income, educational level, tumor staging, complications, and treatment regimen between the anxiety and depression group and the non-anxiety and depression group. There were no differences (P>0.05) in age, BMI, place of residence, family support, marital status, method of medical expense payment, employment status, coping methods, and SSRS scores between the two groups. See **Table 2**.

**Table 2:** Comparison of clinical data between the anxiety and depression groups and the non-anxiety and depression groups.

Factor	Anxiety and depression group(n=66)	No anxiety and depression group(n=167)	*t*/*χ*^2^	*P*
Age(year)			1.929	0.165
≥60	43(65.15)	124(74.25)		
<60	23(34.85)	43(25.75)		
BMI(kg/m^2^)	23.67 ± 4.30	23.59 ± 4.27	0.129	0.898
Gender			28.339	<0.001
man	23(34.85)	121(72.46)		
woman	43(65.15)	46(27.54)		
Place of residence			0.632	0.426
town	39(59.09)	108(64.67)		
countryside	27(40.91)	59(35.33)		
Economic income (10,000 yuan/year)			15.207	<0.001
≥5	25(37.88)	110(65.87)		
<5	41(62.12)	57(34.13)		
Family support situation			0.203	0.652
support	35(53.03)	94(56.29)		
Not supported	31(46.97)	73(43.71)		
Marital status			0.138	0.710
married	45(68.18)	118(70.66)		
Unmarried/divorced/widowed	21(31.82)	49(29.34)		
Educational level			12.223	<0.001
Junior high school and below	42(63.64)	64(38.32)		
High school and above	24(36.36)	103(61.68)		
Payment methods for medical expenses			0.572	0.449
No health insurance	16(24.24)	33(19.76)		
Medical Health Insurance	50(75.76)	134(80.24)		
Working conditions			0.124	0.725
incumbency	40(60.61)	97(58.08)		
retire	26(39.39)	70(41.92)		
Tumor staging			24.563	<0.001
I~II	19(28.79)	108(64.67)		
III	47(71.21)	59(35.33)		
Complication			14.095	<0.001
Yes	42(63.64)	61(36.53)		
No	24(36.36)	106(63.47)		
Treatment regimen			24.648	<0.001
MPT scheme	44(66.67)	52(31.14)		
VDT scheme	22(33.33)	115(68.86)		
Coping			0.045	0.833
Be proactive	35(53.03)	86(51.50)		
Negative coping	31(46.97)	81(48.50)		
SSRS Score (points)			0.107	0.743
<40	32(48.48)	77(46.11)		
≥40	34(51.52)	90(53.89)		

### 3.4 Logistic regression analysis of anxiety and depression in MM chemotherapy patients

Taking whether MM chemotherapy patients had anxiety or depression as the dependent variable (yes=1, no=0), and gender(female=1, male=0), economic income(<50,000 yuan/year=1,≥50,000 yuan/year=0), educational level(below high school=1, high school or above=0), tumor staging(Stage I–II=0, Stage III–IV=1), complications(yes=1, no=0), and treatment regimen(VDT regimen=0, MPT regimen= 1) as independent variables for analysis. The results of the multivariate logistic regression analysis show that gender (OR:5.215, 95%CI:1.78915.202), educational level (OR:13.381, 95%CI:4.45140.228), tumor staging (OR:4.666, 95%CI:1.46814.837), complications (OR:7.063, 95%CI:2.52419.763), and treatment regimen (OR:3.629, 95%CI:1.20710.905) were risk factors for anxiety and depression in MM chemotherapy patients (P<0.05), and economic income (OR:0.578, 95%CI:0.4260.785) was a protective factor (P<0.05). See **Table 3**.

**Table 3:** Logistic regression analysis of anxiety and depression in patients undergoing MM chemotherapy.

Variable	β value	SE value	Wald *χ*^2^ value	*P* value	OR value	95%CI
Gender	1.651	0.546	9.152	0.002	5.215	1.789~15.202
Economic income	-0.548	0.156	12.348	<0.001	0.578	0.426~0.785
Educational level	2.594	0.562	21.333	<0.001	13.381	4.451~40.228
Tumor staging	1.540	0.590	6.812	0.009	4.666	1.468~14.837
Complication	1.955	0.525	13.864	<0.001	7.063	2.524~19.763
Treatment regimen	1.289	0.561	5.270	0.02	3.629	1.207~10.905
Constant	-5.163	0.708	53.109	<0.001	0.006	–

### 3.5 Establishment of a nomogram model for anxiety and depression in MM chemotherapy patients

**Figure 2:**
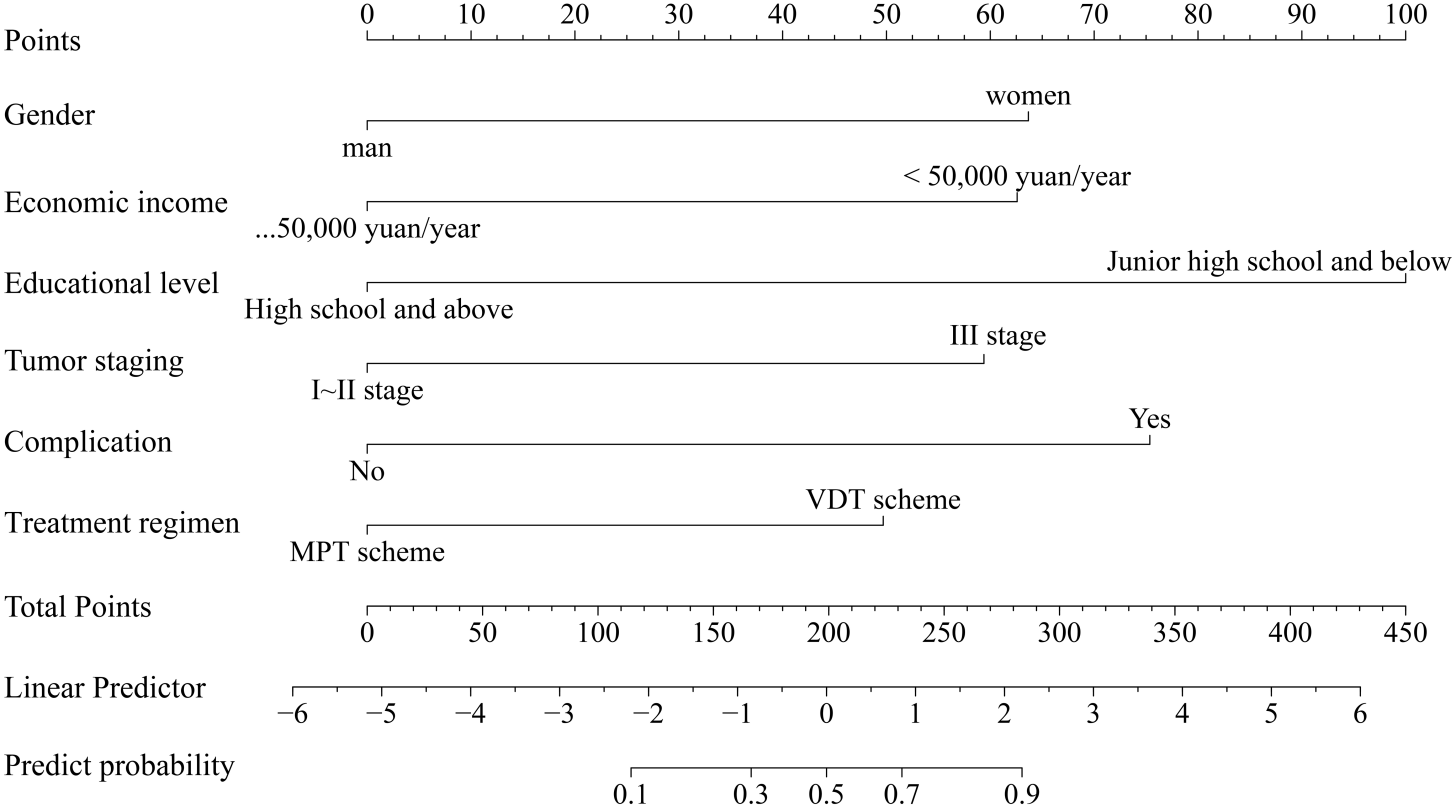
Nomogram for predicting anxiety and depression in MM chemotherapy patients.

The identified risk factors were introduced into R software to establish a nomogram model for predicting anxiety and depression in MM chemotherapy patients. The nomogram was constructed to visually quantify the contribution of each independent factor to the predicted risk. Each variable is assigned a specific number of points: educational level contributes the most, followed by complications, gender, economic income, tumor staging, and treatment regimen. By summing the scores of each variable and calculating the total score, the risk of anxiety and depression in MM chemotherapy patients can be predicted. It is evident that in this model, the most important factor affecting the score is the level of education, followed by complications, gender, economic income, tumor staging, and treatment regimen. **Figure 2** displays the nomogram.

### 3.6 Internal validation of the nomogram model for anxiety and depression in MM chemotherapy patients

**Figure 3:**
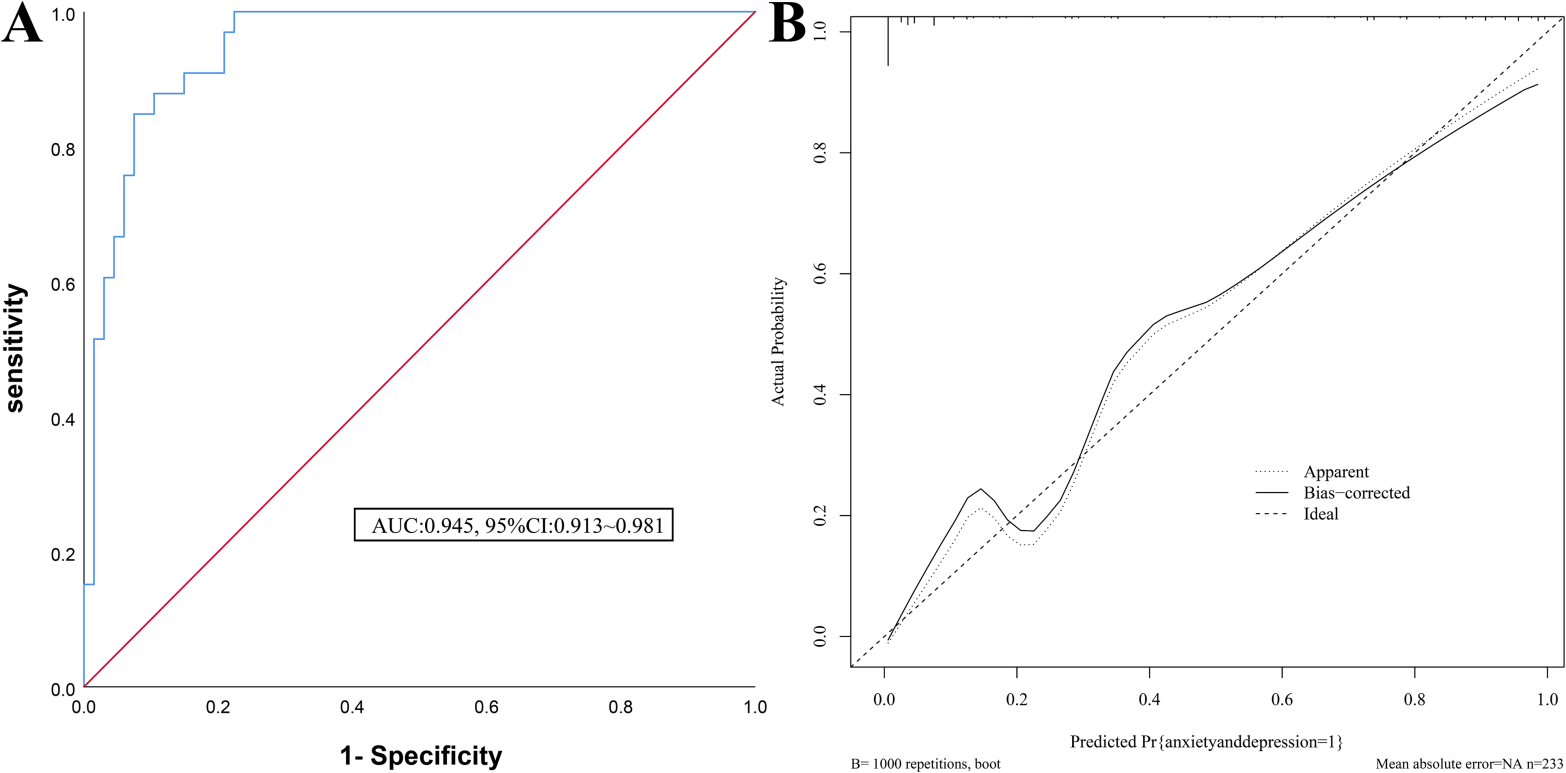
Internal validation of the anxiety and depression nomogram model in patients with MM chemotherapy **(A)** ROC curves of the modeling group; **(B)** Calibration curves of the modeling group.

For the modeling group, the Area Under the Curve (AUC) of the ROC curve was 0.945 (95%CI: 0.913~0.981) (see **Figure 3A**). The calibration curve slope was close to 1 (see **Figure 3B**), and the Hosmer-Lemeshow (H-L) test yielded a *χ*^2^=8.579, P = 0.674, indicating good consistency.

### 3.7 External validation of the nomogram model for anxiety and depression in MM chemotherapy patients

**Figure 4:**
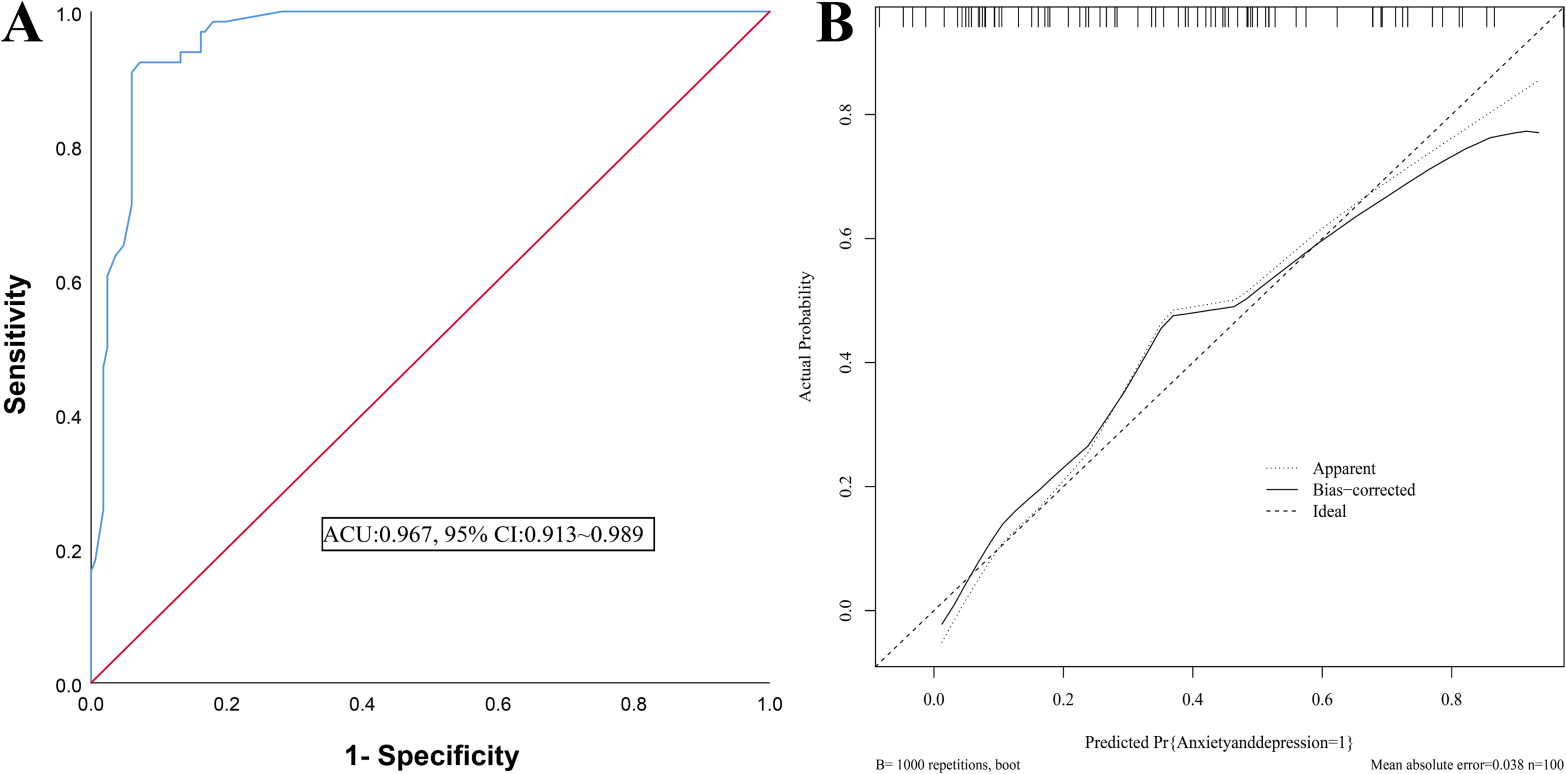
External validation of the anxiety and depression nomogram model in patients with MM chemotherapy **(A)** ROC curves of the verify group; **(B)** Calibration curve of the verification group.

The AUC for external validation was 0.967 (95% CI: 0.913~0.989) (**Figure 4A**). The calibration curve slope was close to 1 (**Figure 4B**), and the H-L test yielded a *χ*^2^=7.315, P = 0.698, indicating good consistency.

### 3.8 DCA curve of the nomogram model

**Figure 5:**
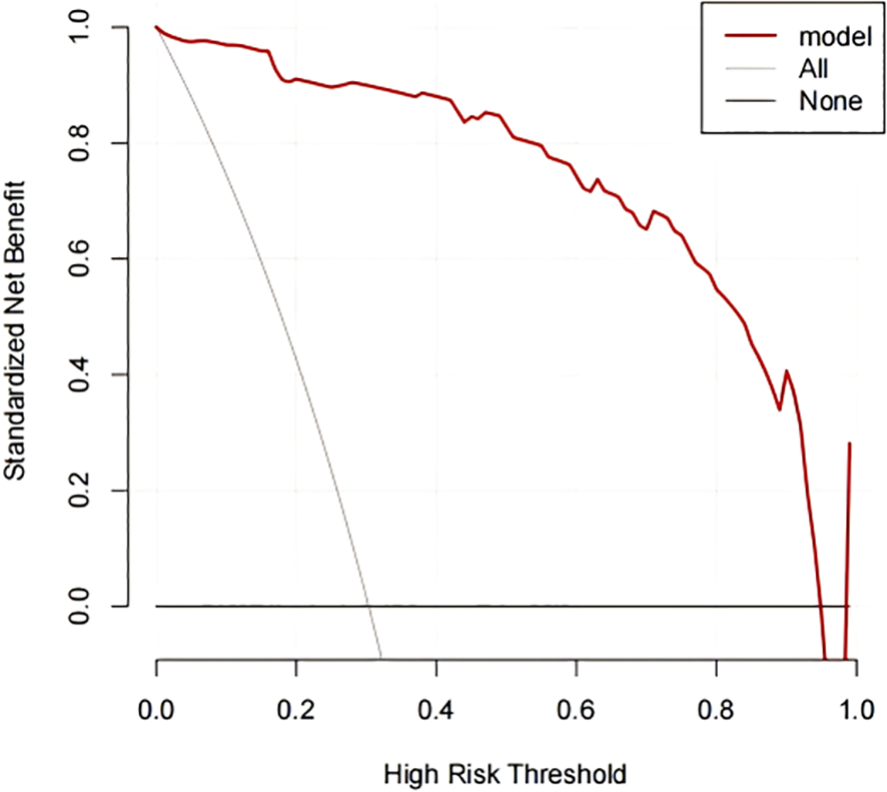
DCA curves for nomogram model. The X-axis represents the continuum of potential threshold probabilities for early recurrence, and the Y-axis represents the net benefit. The red line indicates the nomogram model predicting anxiety and depression in MM chemotherapy patients. The gray line represents the strategy of intervening in all patients, while the thin black line represents the strategy of intervening in none.

As observed from the DCA curve, when the probability ranges from 0.05 to 0.95, the nomogram provided high clinical utility for assessing anxiety and depression risk in MM chemotherapy patients (**Figure 5**).

## 4 Discussion

The incidence of MM is increasing with the aggravation of aging. With the advancement in medical standards and the continuous development of new drugs, the survival period of MM patients has been extended, but this can also create additional physical and psychological burdens (11). Apart from the disease itself, chemotherapy drugs can cause adverse effects such as fatigue and hair loss in patients. Moreover, the proliferation of plasma cells in the body secretes a large number of cytokines, activating osteoclasts and leading to bone pain and limited mobility in patients. Such physical discomfort and restricted activity easily lead to anxiety and depression in patients (12, 13). Therefore, it is important to pay attention to the emotional state of patients during clinical treatment to effectively improve their prognosis. The results of this study show that the incidence of anxiety and depression in MM patients is 28.33%, and the presence of anxiety and depression severely affects the psychological state of patients. Thus, constructing a risk prediction model for anxiety and depression in MM chemotherapy patients is very important for clinical intervention.

Clinically, risk management for anxiety and depression in MM chemotherapy patients can be strengthened based on risk factors. This study identified six influencing factors: gender, economic income, educational level, tumor staging, complications, and treatment regimen. Analysis: ① Compared with male patients, female patients-due to a more family-centered role orientation and potentially lower psychological tolerance-are more likely to develop adverse emotions after falling ill. They are more emotional, and the side effects of chemotherapy, such as endocrine disorders, hair loss and diarrhea, can exacerbate feelings of anxiety and depression (14, 15). ② MM requires long-term treatment, and the high cost of medical care adds to the family burden. Patients may also worry about becoming a burden to their family, leading to anxiety and depression, especially when facing elderly spouses or adult children occupied with work (16, 17). ③ Patients with higher educational levels can more thoroughly understand educational content, have stronger learning abilities, and can acquire treatment-related knowledge through various channels. By contrast, patients with a lower level of education have less ability to understand disease information and may not fully understand health education provided by medical staff, leading to increased anxiety (18, 19). ④ Patients with advanced tumor stages and complications may worry about the incurability of their condition due to disease progression. Complications can exacerbate the disease, and adjustments in the treatment regimen can increase drug dosage and side effects, further aggravating physical discomfort and causing anxiety and depression (20–22). ⑤ The VDT treatment regimen based on Bortezomib can effectively delay the growth of MM. After treatment, it has cytotoxic effects on tumor cells, inhibits the proteasome, and blocks the degradation of target cells through the ubiquitin-proteasome pathway, reducing the adhesion and secretion of tumor cells, leading to their death. Combined with Dexamethasone and Thalidomide, it increases the synergistic effect, inhibits the proliferation of myeloma cells, prolongs survival, and may help alleviate anxiety and depression (23, 24). Nursing interventions can be implemented to address the above influencing factors. During health education, use simple and easy-to-understand language, provide examples of successful cases to increase treatment confidence, and for patients experiencing financial pressure, introduce social assistance programs to alleviate economic burdens.

The nomogram transforms complex regression equations into visual representations, making the results of the predictive model more intuitive and facilitating clinicians’ assessment of patients. In this study, the constructed nomogram yielded AUC values of 0.945 and 0.967 for the two groups, with good fit in the H-L test, indicating strong predictive capability. Additionally, the DCA curve showed that when the high-risk threshold probability ranged from 0.05 to 0.95, the nomogram model had high clinical utility. This model can assist healthcare professionals in predicting the risk of anxiety and depression in MM chemotherapy patients based on risk factors and enable early intervention.

In conclusion, gender, educational level, tumor staging, complications, and treatment regimen are risk factors for anxiety and depression in MM chemotherapy patients, while economic income is a protective factor. The constructed nomogram model has good discriminative ability and consistency, and can visually predict the risk of anxiety and depression in MM chemotherapy patients. This study has limitations, including a relatively small sample size, as well as potential self-report bias from patients diagnosed with anxiety and depression, which may limit generalizability. Future studies should expand sample size and optimize diagnostic procedures for further validation.

## Data Availability

The raw data supporting the conclusions of this article will be made available by the authors, without undue reservation.
